# Re-induction using whole cell melanoma vaccine genetically modified to melanoma stem cells-like beyond recurrence extends long term survival of high risk resected patients - updated results

**DOI:** 10.1186/s40425-018-0456-1

**Published:** 2018-11-29

**Authors:** Jacek Mackiewicz, Tomasz Burzykowski, Dariusz Iżycki, Andrzej Mackiewicz

**Affiliations:** 1 Chair of Medical Biotechnology, University of Medical Sciences, 15 Garbary street, 61-866 Poznan, Poland; 2 Department of Diagnostics and Cancer Immunology, Greater Poland Cancer Centre, 15 Garbary street, 61-866 Poznan, Poland; 3 Department of Medical and Experimental Oncology, Heliodor Świecicki University Hospital, Poznan University of Medical Sciences, Poland 15, 16/18 Grunwaldzka St, 60-780 Poznan, Poland; 4 Department of Biology and Environmental Studies, University of Medical Sciences, 8 Rokietnicka street, 60-806 Poznan, Poland; 5 Interuniversity Institute for Biostatistics and statistical Bioinformatics, Hasselt University, 42 Martelarenlaan street, 3500 Diepenbeek, Belgium; 6 BioContract Sp z o.o., 36 Zambrowska street, 61-051 Poznan, Poland

**Keywords:** Melanoma, Immunotherapy, Genetic melanoma vaccine, Long-term survivals, Phase II clinical trials, Re-induction

## Abstract

**Background:**

AGI-101H is an allogeneic gene modified whole cell therapeutic melanoma vaccine, evaluated in over 400 melanoma patients in the adjuvant and therapeutic settings. We present updated long-term survival results from two single-arm, phase II adjuvant trials (Trial 3 and Trial 5) with the focus on treatment beyond recurrence of the disease.

**Methods:**

Patients with resected high-risk melanoma (stage IIIB-IV) were enrolled to Trial 3 (*n* = 99) and Trial 5 (*n* = 97). The primary endpoint was disease-free survival (DFS), and the secondary was overall survival (OS). In the induction phase, the vaccine was administered every 2 weeks (eight times), followed by the maintenance phase every month until progression. At progression, maintenance was continued or re-induction was applied with or without surgery.

**Results:**

In Trial 3, the 10-year DFS was equal to 33.0% overall and to 52.4, 25.0, and 8.7% for stage IIIB, IIIC, and stage IV patients, respectively. In Trial 5, the overall 10-year DFS was equal to 24.2%, and to 37.5, 18.0, and 17.6% for stage IIIB, IIIC, and stage IV patients, respectively. In Trial 3, the 10-year OS was equal to 42.3% overall, and to 59.5, 37.5, and 17.4% for stage IIIB, IIIC, and stage IV patients, respectively. In Trial 5, the 10-year OS was equal to 34.3% overall and to 46.9, 28.0, and 29.4% for stage IIIB, IIIC, and stage IV patients, respectively. Among the 65 patients of Trial 3 who developed progression, 43 received re-induction with (*n* = 22) or without (*n* = 21) surgery. Two patients received surgery without re-induction. All the 22 progressing patients, who did not receive re-induction, died. Among the 75 patients of Trial 5 who experienced progression, 39 received re-induction with (*n* = 21) or without (*n* = 18) surgery. Among the 36 progressing patients who did not receive the re-induction, 35 died. Surgery and re-induction reduced (independently) the increase of mortality after progression in both trials, with the effect of re-induction reaching statistical significance in Trial 5.

**Conclusions:**

Vaccination beyond recurrence of the disease with additional re-induction combined with surgery or alone increased long term survival of melanoma patients. However, further studies on larger patient cohorts are required.

**Trial registration:**

Central Evidence of Clinical Trials (EudraCT Number 2008–003373-40)

**Electronic supplementary material:**

The online version of this article (10.1186/s40425-018-0456-1) contains supplementary material, which is available to authorized users.

## Introduction

Recently, a significant progress has been made in the treatment of patients with melanoma. The approval of anti-PD1 (nivolumab, pembrolizumab) and BRAF and MEK inhibitors (darbafenib plus trametinib and vemurafenib plus cobimetinib) changed the treatment landscape in advanced melanoma [1, 2]. Moreover, ipilimumab (anti-CTLA4) and nivolumab were recently approved by the U.S. FDA (United States Food and Drug Administration) in the treatment of patients after resection of high-risk melanoma. Also dabrafenib and trametinib reached marketing authorization in this indication. However, there are still limitations related to the safety, acquired resistance and balance between toxicity and effectiveness. Moreover, there are no data yet on the long-term survival of treated patients. An intensive research is conducted on basic and clinical levels to increase the efficacy and reduce toxicity of the aforementioned treatment modalities. Cancer researchers are working on better understanding of mode of action, resistance and transient effects on the basic levels, while oncologists are testing combinations of various treatment strategies in clinical trials [3–7].

AGI-101H is an allogeneic gene modified whole cell therapeutic melanoma vaccine [8]. Gene modification of the vaccine cells has changed their phenotype towards melanoma stem cells-like (MSC) [9]. Immunization of patients generated anti-MSC cellular and humoral immune responses leading to immune - targeting of MSC. AGI-101H was evaluated in four single-arm phase II studies in over 400 patients with resected or non-resectable metastatic melanoma demonstrating high efficacy. We have earlier reported results of two phase II studies – Trial 3 (*n* = 97) and Trial 5 (*n* = 99) in patients with resected high-risk melanoma (stage form IIIB to IV-M1c). Patients were immunized with AGI-101H every 2 weeks (induction phase) for 4 months, then every month (maintenance) until death. At progression of the disease, based on investigators decision, induction phase was repeated (re-induction) and followed by maintenance. Re-induction was applied alone or in combination with surgery. We observed high objective response rate (Trial 3: 24%, Trial 5: 26%) and stable disease (Trial 3: 33%, Trial 5: 5%) in patients receiving re-induction only, followed by maintenance. Re-induction with or without surgery was associated with reduced hazard of disease progression. The 5-year overall survival (OS) of patients in Trial 3 was as follows: stage IIIB – 66.7%, IIIC – 43.8%, IV – 26.1 (10-year OS: IIIB – 59.5%, IIIC – 37.5%, IV – 17.4%), while in Trial 5: IIIB – 56.3%, IIIC – 39.8%, IV – 41.2%. No grade 3–4 toxicity was noted [10].

In this paper we report updated results (disease-free survival, DFS; overall survival, OS; and outcomes of patients receiving re-induction with AGI-101H) of these two phase II studies (Trial 3 and Trial 5). In November 2008 all alive patients were transferred into one trial “Extended Treatment for Advanced Melanoma Patients Transferring from Trials 2-5” (EnduraCT Number: 2008–003373-40) with a Study Objective: To determine the long term safety profile including survival of AGI-101H administered s.c. for extended use.

## Methods

Study design, inclusion and exclusion criteria, vaccine composition, treatment scheme, tumor assessment criteria after disease progression, toxicity assessment and frequency of follow-up were described earlier [10] and are further included in Additional file 1. DFS time was computed from the beginning of the vaccination treatment until the date of first progression or death (complete observations) or the date of the last observation (censored observation) before September 8, 2017. OS time was computed from the beginning of the vaccination treatment until death (complete observations) or the date of the last observation (censored observation) before September 8, 2017. In patients receiving re-induction, post-re-induction OS was computed from the date of the start of re-induction to death or the last observation.

DFS and OS functions were estimated by the Kaplan-Meier method. Confidence intervals (CI) for survival probabilities were computed by using the log-log transformation of the estimates of the probabilities. Median survival times were estimated based on the estimated survival functions [10]. Confidence intervals for the median survival times were estimated based on the upper and lower limits of the confidence intervals of survival probabilities.

The effect of re-induction was assessed by estimating the hazard ratio (HR) based on a Cox model for OS with time-dependent binary covariates [10] for progression (indicating when the progression occurred), surgery (indicating when a surgical resection of new lesions was applied), and re-induction (indicating when the re-induction was started).

The median follow-up time was estimated by using the “reverse” Kaplan-Meier method, i.e., by treating deaths as censored observations.

All computations were performed by using STATA v.13 (StataCorp LP, College Station, TX). Statistical significance tests were assessed by using the 5% significance level (two-sided).

## Results

The detailed patients’ enrollment and characteristics at baseline (Additional file 1: Table S1) and at first progression (Additional file 1: Table S2) were described elsewhere [10]. In Trial 3, the median follow-up time at data cutoff on September 8, 2017 was equal to 17.0 years (enrollment from 1996 till 2001). In Trial 5 the median follow-up time was equal to 12.9 years at data cutoff on September 8, 2017 (enrollment from 2002 till 2005).

### Disease-free survival (DFS)

In Trial 3, progression of the disease was observed in 65 patients. Six patients died without progression. Thus, there were 71 events in total. The median DFS time was estimated to be equal to 1.8 years (95% C.I.: [1.2, 2.7]).

**Fig. 1:**
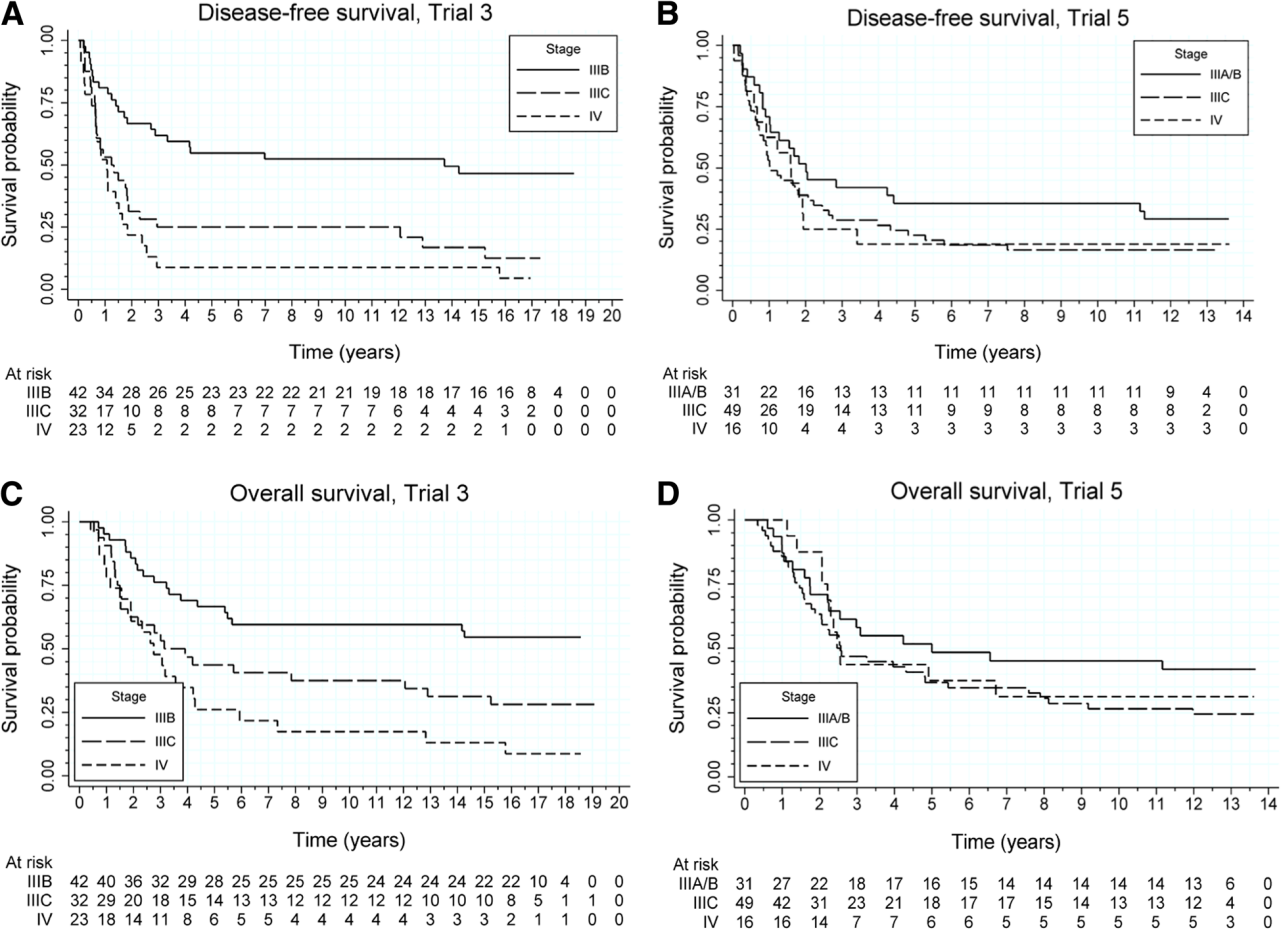
Disease-free survival and overall survival in treated patients. Disease-free survival according to stage of the disease in melanoma patients treated with AGI-101H in Trial 3 (**a**) and Trial 5 (**b**). Overall survival according to stage of the disease in melanoma patients treated with AGI-101H in Trial 3 (**a**) and Trial 5 (**b**)

Figure 1a presents the estimated DFS curves according to the stage of disease. The estimated median DFS time for stage IIIB was equal to 13.7 years (lower 95% C.I. limit: 2.7 years); for stage IIIC and IV, the median DFS time was estimated to be equal to 1.2 years (95% C.I.: [0.6, 1.9]) and 1.1 years (95% C.I.: [0.6, 1.5]), respectively. The estimated probability of surviving 10 years without recurrence of the disease was equal to 33.0% overall, and to 52.4, 25.0, and 8.7% for stage IIIB, stage IIIC, and stage IV patients.

In Trial 5, progression of the disease was observed in 75 patients. Two patients died without progression. Thus, there were 77 events in total. The median DFS time was estimated to be equal to 1.6 years (95% C.I.: [1.0, 2.0]).

Figure 1b presents the estimated DFS curves according to the stage of disease. The median DFS time was estimated to be equal to 2.0 years (95% C.I.: [1.0, 11.2]) for stage IIIB (2 IIIA patients included), to 1.0 years (95% C.I.: [0.7, 2.1]) for stage IIIC, and to 1.6 years (95% C.I.: [0.6, 1.9]) for stage IV. The estimated probability of surviving 10 years without recurrence of the disease was equal to 24.2% overall, while was 37.5, 18.0, and 17.6% for stage IIIA/B, stage IIIC, and stage IV patients, respectively.

### Overall survival (OS)

In Trial 3, death was noted in 63 patients. At the time of data analysis, 32 patients were alive; two patients were lost to follow-up before data cutoff. The median OS time was estimated to be equal to 4.4 years (95% C.I.: [3.1, 12.8]). Figure 1c presents the estimated OS curves according to the stage of disease. The lower 95% C.I. limit for the median OS time for stage IIIB was equal to 5.4 years; for stage IIIC and IV, the median OS time was estimated to be equal to 3.1 years (95% C.I.: [1.5, 12.9]) and 2.7 years (95% C.I.: [1.6, 4.2]), respectively. The estimated probability of surviving 10 years was equal to 42.3% for all patients, and to 59.5, 37.5, and 17.4% for stage IIIB, stage IIIC, and stage IV patients, respectively.

In Trial 5, death was noted in 67 patients. At the time of data analysis 32 patients were alive. The median OS time was estimated to be equal to 3.1 years (95% C.I: [2.3, 5.4]). Figure 1d presents the estimated OS curves according to the stage of disease. The median OS time for stage IIIB was estimated to be equal to 5.0 years (lower 95% C.I. limit: 2.2 years); for stage IIIC and IV, the median survival time was estimated to be equal to 2.6 years (95% C.I.: [1.9, 4.8]) and 2.5 years (lower 95% C.I. limit: 2.1), respectively. The estimated probability of surviving 10 years was equal to 34.3% overall, while was 46.9, 28.0, and 29.4% for stage IIIA/B, stage IIIC, and stage IV patients, respectively.

### Re-induction

**Fig. 2:**
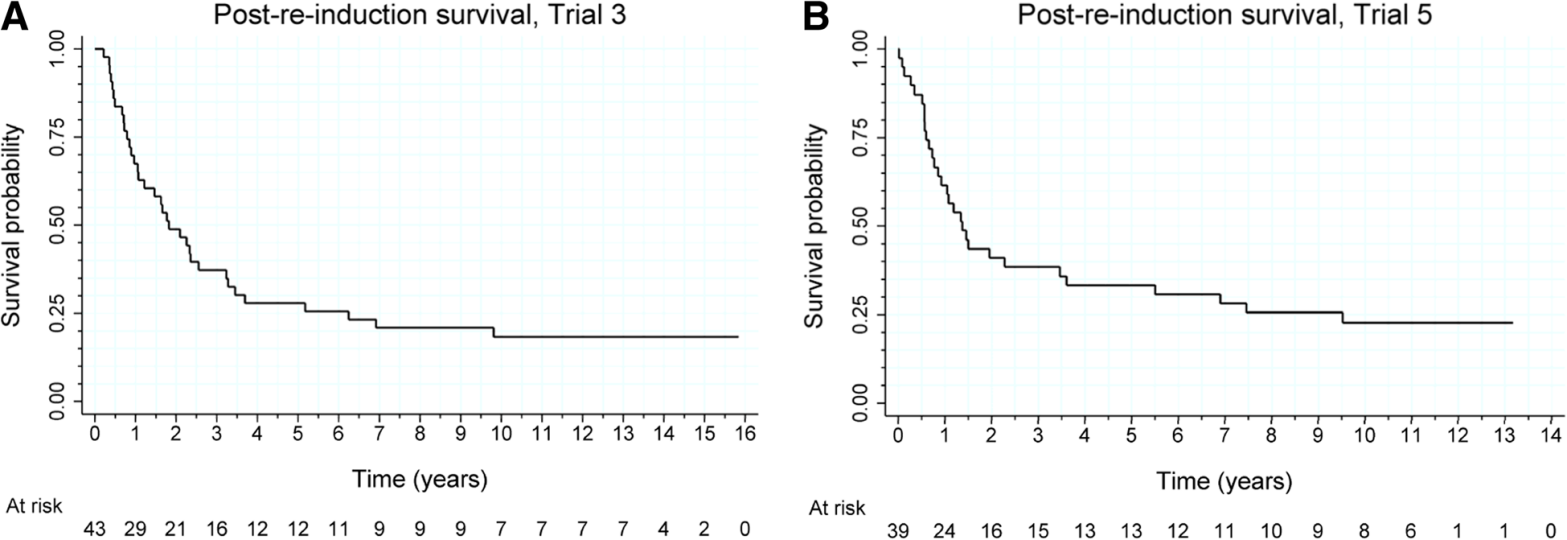
Overall survival of patients treated with AGI-101H re-induction in Trial 3 (**a**) and Trial 5 (**b**)

In Trial 3 re-induction was applied in 43 (66.1%) of the 65 patients, who experienced progression after the initial vaccination therapy. All the 22 progressing patients, who did not receive re-induction, died. Among the 43 patients treated with re-induction, seven were alive at data cutoff; one was lost to follow up on August 20, 2015. Figure 2a presents survival time for patients treated with re-induction, computed from the date of initiation of the re-induction therapy. The estimated median OS time was equal to 1.8 years (95% C.I.: [1.0, 3.2]). Among the 65 patients, who experienced progression, 24 (36.9%) were treated surgically. In two cases, surgery took place at progression, while in the remaining cases post-progression. Surgery was applied in 22 (51.1%) of the 43 patients who received re-induction. In 14 cases, surgery was carried out before the initiation of re-induction (mean: 159 days; median: 47 days); in one case at the date of the start of re-induction, while in seven cases after the start of re-induction.

The effect of re-induction on patients’ survival was analyzed by the Cox model with time-dependent covariates indicating the occurrence of progression, the use of surgery, and the initiation of the re-induction. The estimated HR for progression was equal to 41.5 (95% C.I.: [15.9, 108.3]; *p* < 0.001), for re-induction it was equal to 0.58 (95% C.I.: [0.31, 1.08]; *p* = 0.09), and for surgery it was equal to 0.48 (95% C.I.: [0.26, 0.88]; *p* = 0.02). Thus, while progression increased the mortality hazard about 42 times, surgery and re-induction reduced the increase (independently of each other) by about 52 and 42%, respectively; thus, the combined reduction due to the joint effect of surgery and re-induction can be estimated to be equal to (1–0.58∙0.48)∙100% = 72%. Note, however, the effect of re-induction was statistically non-significant.

In Trial 5, re-induction was used in 39 (52.7%) of the 75 patients, who experienced progression after the initial vaccination therapy. Among the 36 progressing patients, who did not receive the re-induction, 35 died. Among the 39 patients treated with re-induction, nine were alive at data cutoff.

Surgery was applied in 21 (72.4%) of the 39 patients who received re-induction. In 18 cases, surgery took place before the start of re-induction (mean: 49.6 days; median: 26 days), while in three cases at the date of the start of re-induction. Figure 2b presents survival time of patients treated with re-induction, computed from the date of initiation of the re-induction therapy. The estimated median OS time is equal to 1.4 years (95% C.I.: [0.8, 3.6]).

The effect of re-induction on patients’ survival was analyzed by the Cox model with time-dependent covariates indicating the occurrence of progression, the use of surgery, and the initiation of the re-induction. The estimated HR for progression was equal to 63.5 (95% C.I.: [24.0, 168.4]; *p* < 0.001), for re-induction it was equal to 0.34 (95% C.I.: [0.18, 0.63]; *p* = 0.001), and for surgery it was equal to 0.57 (95% C.I.: [0.32, 1.02]; *p* = 0.06). Thus, while progression increased the mortality hazard about 60 times, surgery and re-induction reduced the increase (independently of each other) by about 43 and 66%, respectively; thus, the combined reduction due to the joint effect of surgery and re-induction can be estimated to be equal to (1–0.34∙0.57)∙100% = 81%. Note that the effect of re-induction was statistically significant.

## Discussion

The adjuvant treatment landscape in resected melanoma patients is changing rapidly. The approval of ipilimumab, nivolumab and dabrafenib combined with trametinib in this indication, demonstrates very high potential of these drugs. However, ipilimumab displayed very high toxicity and failed to obtain approval in Europe [11]. Moreover, physicians are very cautious with prescribing ipilimumab in the adjuvant setting and other less toxic and more effective therapies like nivolumab and perhaps pembrolizumab are warranted. Currently a phase III adjuvant study evaluating the combination of ipilimumab (low dose) and nivolumab in high-risk melanoma patients is ongoing [12]. Recently, Wang and colleagues have reported skin reactions associated with anti-PD1 therapy such as psoriasis, lupus, sarcoidosis, eczema in 40% of patients. The onset of the adverse events varied, however in one-third of patients occurred when the applied treatment period was terminated [13].

AGI-101H is an allogeneic whole cell based vaccine modified with a gene encoding hyper-interleukin-6 (H6) [14, 15]. Such genetic modification led to conversion of vaccine allogeneic melanoma cells into melanoma stem cells (MSC)-like. Others have confirmed later that activation by interleukin IL-6 (IL-6) of JAK1-STAT3-OCT4 pathway may convert differentiated breast cells (non-cancer stem cells) into breast cancer stem cells [16]. However, chronic exposure of cells caring membrane receptor (IL-Rα, gp80, IL-6 binding protein) to IL-6 leads to desensitization of these cells to IL-6 due to the down-regulation of IL-6Rα. The responsiveness of these cells to IL-6 may be restored by soluble IL-6Rα (sIL-6R) [17]. IL-6/sIL-6R soluble complex directly targets gp130 (signal transducing receptor subunit β) and activates cells, which do not posses IL-6Rα via *transsignaling*. Fusion of IL-6 with agonistic sIL-6R assembled via an artificial linker on cDNA level, resulted in generation of transgenic fusion protein, which is stable and displays 10–1000 fold higher activity than soluble IL-6/sIL-6R complex in various biological systems [18]. Accordingly, the new fusion protein was referred to as supercytokine or hyper-IL-6 (H6). The vaccine composition and mechanism of action of AGI-101H were published elsewhere [10, 19–23], however, recently we have demonstrated specific immunotargeting of MSC [9]. In circulation of long-term surviving patients we found aldehyde dehydrogenase isoenzyme (ALDH1A1) directed cytotoxic central memory T CD8^+^ cells and specific anti-ALDH1 antibodies. These immune response tests were done in long-term survival melanoma patients treated with AGI-101H in Trial 3 and Trial 5. The results show that vaccination can activate the recipient’s immunity against the vaccine [9]. In various experimental models immunotargeting of ALDH1A1 resulted in cancer stem cells eradication, including MSC [24].

Results presented in the current report are unique due to very long follow-up and continuation of the treatment for several years. The strategy of AGI-101H re-induction with or without surgery is beneficial for the patients and needs further validation in planned phase III randomized, multi-center study with anti-PD1 in the control arm and identified companion biomarker evaluation. An important advantage of AGI-101H therapy is its very low toxicity. With over 400 patients treated and 30,000 administered doses of the vaccine, no treatment-related grade 3–5 toxicities were observed. The adverse events associated with the vaccine were mostly limited to local reactions at the injection site of grade 1 or 2. Some of the patients developed arthralgia or elevated body temperature ([10, 25], data unpublished).

AGI101H is a very good candidate for combinational treatment with immune check-point inhibitors like anti-PD1/PD-L1 or others in the adjuvant or metastatic setting. Immune check-point synapses are not cancer specific. Accordingly, combination of specific immuno-targeting of MSC with inhibitors of immune-check points may enhance the effectiveness of both therapies. It is supported by our studies showing that surgery of recurring metastases during adjuvant treatment eliminates tumor-induced immunosuppression and prevents further progression. Number of preclinical studies demonstrated synergistic effect of whole cell based vaccines combined with immune-checkpoint inhibitors [26–36]. Although clinical trials evaluating cellular vaccines with immune-check point inhibitors are still rare, early phase studies demonstrated high potential of such strategy [37–39]. Some early phase trials evaluating multi-peptide vaccines combined with immune-check point inhibitors were performed, demonstrating promising results [40, 41], eg. multi-peptide vaccine (gp100, MART-1, NY-ESO-1) plus nivolumab showed 1-year survival in 87% of resected melanoma patients [41].

Various reports demonstrated clinical benefit from continuation of the treatment with anti-PD1 beyond progression of the disease in advanced melanoma patients [42, 43]. In the analysis of two phase III studies (Check-Mate 066/067), the response rate in patients treated with nivolumab beyond progression was 28%, with another 16% of patients developing stable disease with a reduction of tumor burden. These responses were durable. These observations suggest that some patients might benefit from treatment beyond progression, however further studies are needed to select patients that might benefit most from such strategy [43]. In our phase II study conducted in metastatic melanoma (Trial 2), the response rate in patients treated with AGI-101H beyond progression with re-induction was equal to 46%, showing high benefit from treatment continuation with intensified treatment dosing [25]. Treatment with AGI-101H beyond recurrence of the disease with additional re-induction in resected melanoma patients was linked to high response rate (Trial 3: 24%, Trial 5: 26%) [10]. In the currently presented analysis we observed that treatment beyond recurrence with initial re-induction (8 doses of AGI-101H every 2 weeks), and followed by maintenance every 4 weeks was linked with reduction of mortality hazard in both Trial 3 and Trial 5, though it reached statistical significance only in Trial 5. These observations show that some patients might benefit from treatment beyond recurrence/progression with additional re-induction. The conclusion that can be drawn from this study is that the highest benefit is observed in patients who received re-induction combined with surgical resection of the recurrence. However, this approach was applied in patients with operable recurrent disease. It is known that melanoma patients benefit from the resection of metastases, while addition of vaccine re-induction enhances the survival benefit. Patients with inoperable disease received only re-induction without surgery. Re-induction in these patients was linked with high response rate translating into survival benefit, while all patients (excluding one individual in Trial 5) not receiving re-induction died. However, to define a group of patients benefiting most from such strategies, further evaluation in a randomized study is needed, including biomarker validation studies.

We have identified blood pretreatment epigenetic prognostic biomarkers associated with favorable survival of patients with resected disease treated with AGI-101H in the adjuvant setting. It is possible that these markers reflect status of the host immune system. We are currently studying the role and mechanisms of action of these biomarkers in mounting of the anti-melanoma immune response, before we publish the results.

The main limitation of the analyzed studies is that they are single arm trials conducted in a single institution, which may make them vulerable to selection bias. Also, in case of recurrence, the decision on the use of re-induction and/or surgery was not based on randomization, but left to the investigator.

However, the reported findings based on the two studies are very important for designing future immunotherapy trials, involving blockade of immune-check point synapses, as well as trials combining the blockade with cancer vaccines. Treatment beyond recurrence/progression with intensified vaccine dosing (re-induction) and/or surgery may overcome resistance to the treatment, translating into patients benefit. Future immunotherapy (anti-PD1/PD-L1 + cellular cancer vaccines) trial designs in the adjuvant setting should include an option of complete resection of recurring disease combined with treatment continuation with intensified dosing of the vaccines and anti-PD1/PD-L1 continuation. While patients with inoperable recurrence should receive vaccine re-induction combined with anti-PD1/PD-L1 therapy continuation or they should be offered a change of therapy regimen especially if a reasonable further treatment options exist. Furthermore, the vaccination treatment period should not be limited (eg. to one year) in patients treated in the adjuvant setting, especially in those without significant treatment related adverse events. In these patients continuation of vaccination till clinical benefit is observed should be applied. However, the economic aspect of adjuvant treatment elongation might pose a challenge.

## Additional file

Additional file 1: Study design, Procedures, Study endpoints and Patients characteristics in Trial 3 and Trial 5. (DOCX 23 kb)
