# Supplementary material for: Re-induction using whole cell melanoma vaccine genetically modified to melanoma stem cells-like beyond recurrence extends long term survival of high risk resected patients - updated results
Source: J Immunother Cancer. 2018 Nov 29;6:134. doi: 10.1186/s40425-018-0456-1 (PMC6264600; doi:10.1186/s40425-018-0456-1)
Supplement: Supplementary file 1 — Study design, Procedures, Study endpoints and Patients characteristics in Trial 3 and Trial 5. (DOCX 23 kb) [file 40425_2018_456_MOESM1_ESM.docx]

Additional file 1

Additional file methods

**Study design and patients**

These two phase two clinical trials (Trial 3 and Trial 5) are single-arm, open-label, single-institution studies. Enrolment criteria in Trial 3 and Trial 5 were identical and included, patients >18 years old with histologically confirmed stage IIIB, IIIC, IV melanoma with complete surgical resection of all clinically evident metastases. Previous chemotherapy or immunotherapy had to be completed at least 4 weeks before enrolment. Inclusion criteria in ETAM (Extended Treatment for Advanced Melanoma) study included participation in one of the previous studies (Trial 2-5) evaluating alternative formulations of the same active substance - genetically modified melanoma cells. Complete eligibility criteria have been previously published [1].

**Procedures**

AGI-101 and AGI-101H vaccines consist of admixture (1:1) of irradiated two allogeneic melanoma cell lines Mich-1 and Mich-2, which were modified with cDNA of designer cytokine Hyper-interleukin 6. Patients were immunized with AGI-101 in Trial 3 and Trial 5 and then with AGI-101H (from November 2008) in ETAM study. The original formulation of AGI-101 cryopreservation medium included fetal calf serum, which in AGI-101H was replaced by human albumin. The details of the cryopreservation medium modifications were previously published [1].

In the induction phase 5 x 10^7^ vaccine cells were administered subcutaneously eight times in two weeks intervals. Next maintenance phase of the vaccine injection every month was applied. In case of disease recurrence, maintenance was continued or re-induction was repeated and followed by maintenance. The re-induction was applied alone or in combination with surgery. At disease recurrence the management was based on the physician decision. [1].

Tumor clinical responses in patients vaccinated beyond recurrence (without surgical resection) were assessed using Response Evaluation Criteria In Solid Tumors (RECIST) [1].

At baseline, patients underwent assessments, which included demographics, medical history, vital signs, WHO performance status, blood laboratory analyses, physical examination, radiological assessment. Physical examination, WHO performance status, vital signs, chemistry, and hematology tests were performed at every study visit (prior to vaccination – every 2 or 4 weeks). Tumor assessment was carried out at weeks 6, 18, and 34 and then every 2 months [1].

**Study endpoints**

The primary endpoint in Trial 3 and Trial 5 was disease-free survival (DFS). The secondary endpoint in both trials was overall survival (OS) [1].

Trial 3 and Trial 5 were approved by the Regional Bioethics Committee (RBC) in Poznań, Poland. ETAM trial was approved by RBC and Central Evidence of Clinical Trials (EudraCT Number 2008-003373-40).

**Additional file 1**

**Additional file 1: Table S1.**

**Additional file 1: Table S2.**

**Table S1. Patients characteristics at baseline**

|  | Trial 3  (n=97) | Trial 5  (n=99) |
| --- | --- | --- |
| Age (mean, ± SD), years | (46.7 ± 12.3) | (50.0 ± 12.4) |
| Male, *n* (%) | 46 (47%) | 48 (48%) |
| Stage, *n* (%)  IIIA  IIIB  IIIC  IV  M1a  M1b  M1c | 0 (0%)  42 (43%)  32 (33%)  23 (24%)  12 (12%)  2 (2%)  10 (10%) | 2 (2%)  30 (30%)  50 (51%)  17 (17%)  14 (14%)  2 (2%)  1 (1%) |
| Prior treatment, *n* (%)  None  Radiotherapy  Radiotherapy  + hormone therapy  Chemotherapy  +/- hormone therapy  Unknown | 75 (77%)  11 (12%)  2 (2%)  7 (7%)  1 (1%) | 67 (68%)  12 (12%)  0 (0%)  4 (4%)  16 (16%) |

**Table S2. Patients characteristics at first progression**

|  | Trial 3  (n=43) | Trial 5  (n=39) |
| --- | --- | --- |
| Male, *n* (%) | 19 (44%) | 17 (44%) |
| Stage, *n* (%)  IIIB  IIIC  IV  M1a  M1b  M1c | 3 (7%)  11 (26%)  29 (67%)  20 (46%)  2 (5%)  7 (16%) | 0 (0%)  7 (18%)  32 (82%)  14 (36%)  2 (5%)  16 (41%) |

**Additional file references**

1. Mackiewicz A, Mackiewicz J, Wysocki PJ, Wiznerowicz M, Kapcinska M, Laciak M, Rose-John S, Izycki D, Burzykowski T, Karczewska-Dzionk A. [Long-term survival of high-risk melanoma patients immunized with a Hyper-IL-6-modified allogeneic whole-cell vaccine after complete resection.](https://www.ncbi.nlm.nih.gov/pubmed/22577889) Expert Opin Investig Drugs. 2012 Jun;21(6):773-83.
